# Image Quality Analysis of Eyes Undergoing LASER Refractive Surgery

**DOI:** 10.1371/journal.pone.0148085

**Published:** 2016-02-09

**Authors:** Samrat Sarkar, Pravin Krishna Vaddavalli, Shrikant R. Bharadwaj

**Affiliations:** 1 Prof. Brien Holden Eye Research Centre, Hyderabad Eye Research Foundation, L V Prasad Eye Institute, Hyderabad, 500034, Telangana, India; 2 Cornea and Anterior Segment Services, L V Prasad Eye Institute, Hyderabad, 500034, Telangana, India; 3 Bausch and Lomb School of Optometry, L V Prasad Eye Institute, Hyderabad, 500034, Telangana, India; University of Surrey, UNITED KINGDOM

## Abstract

Laser refractive surgery for myopia increases the eye’s higher-order wavefront aberrations (HOA’s). However, little is known about the impact of such optical degradation on post-operative image quality (IQ) of these eyes. This study determined the relation between HOA’s and IQ parameters (peak IQ, dioptric focus that maximized IQ and depth of focus) derived from psychophysical (logMAR acuity) and computational (logVSOTF) through-focus curves in 45 subjects (18 to 31yrs) before and 1-month after refractive surgery and in 40 age-matched emmetropic controls. Computationally derived peak IQ and its best focus were negatively correlated with the RMS deviation of all HOA’s (HORMS) (r≥-0.5; p<0.001 for all). Computational depth of focus was positively correlated with HORMS (r≥0.55; p<0.001 for all) and negatively correlated with peak IQ (r≥-0.8; p<0.001 for all). All IQ parameters related to logMAR acuity were poorly correlated with HORMS (r≤|0.16|; p>0.16 for all). Increase in HOA’s after refractive surgery is therefore associated with a decline in peak IQ and a persistence of this sub-standard IQ over a larger dioptric range, vis-à-vis, before surgery and in age-matched controls. This optical deterioration however does not appear to significantly alter psychophysical IQ, suggesting minimal impact of refractive surgery on the subject’s ability to resolve spatial details and their tolerance to blur.

## Introduction

LASER Assisted In Situ Keratomileusis (LASIK) and Photorefractive Keratectomy (PRK) have become treatments of choice for correcting myopia and achieving freedom from spectacles and contact lenses until the onset of presbyopia [1]. While the overall myopic refractive error is effectively reduced following surgery, the magnitude of the eye’s higher-order wavefront aberrations (HOA’s; specifically spherical aberrations and coma) increase significantly following refractive surgery, vis-à-vis, the pre-operative values [2–5]. The magnitude of these aberrations subsequently either remains constant post-operatively or decreases only modestly within few months of surgery [2, 3].

Retinal image quality (IQ) and spatial vision (e.g. visual acuity, contrast sensitivity) are significantly affected in the presence of HOA’s [6–11]. Computational through-focus analyses indicate that peak IQ drops and the resultant sub-optimal IQ extend over a larger dioptric range [i.e. an expansion of the depth-of-focus (DOF)] with an increase in HOA’s [11, 12]. Psychophysical observations made by inducing HOA’s in otherwise “normal” eyes also indicate that the logMAR acuity and the refractive endpoint that produces best acuity is dependent on the magnitude and interaction between HOA’s [7, 11, 13]. Psychophysical DOF also widens with an increase in HOA’s, especially with spherical aberrations [12, 14–16]. All these observations suggest that subjects undergoing refractive surgery may also experience similar modifications to their visual experience owing to the increased post-operative HOA’s. Specifically, the peak retinal IQ of these eyes may decline and their DOF may widen post-operatively with an increase in the HOA’s. Peak IQ and DOF may also be negatively correlated with each other in these eyes. The goal of this study was therefore to systematically document the relationship between peak IQ, dioptric plane where peak IQ was achieved (termed as best focus in this study), DOF and the magnitude of HOA’s in subjects who undergo wavefront guided LASIK and PRK procedures. Such a systematic documentation of changes in IQ following refractive surgery has not been performed thus far, although earlier studies on this topic do indicate an overall loss in visual performance with an increase in corneal wavefront aberrations following radial keratotomy or conventional LASIK refractive surgery [17–20].

The study was divided into two arms. In the first arm, all outcome variables were measured in cases after 1-month of uneventful refractive surgery and they were compared with age-matched controls. In the second arm, all outcome variables were obtained from subjects before and 1-month after uneventful refractive surgery. The relation between all outcome variables was determined in this study using computational (Visual Strehl ratio based optical transfer function—VSOTF [21]) and psychophysical (high contrast logMAR acuity) through-focus curves. The comparison of computational and psychophysical data provided a comprehensive understanding of the monocular spatial visual experience of subjects undergoing refractive surgery. The computational analysis estimated the IQ experienced by these eyes in the presence of optical degradation while the psychophysical analysis determined if this change in IQ had any practical consequences on the individual’s visual resolution and blur tolerance. These results also have important implications for near vision following refractive surgery, as blur-driven accommodation and its coupled accommodative vergence may be modulated by the pattern of HOA’s experienced in the two eyes [22–25].

## Materials and Methods

Thirty cases (18–31yrs) and 40 controls (20–28yrs) participated in the first arm and 45 subjects (20–34yrs) participated in the second arm of this study. The two arms of the study were planned sequentially and executed at different time points. Different sets of subjects therefore participated in the two arms of the study. The first arm of the study compared the IQ of cases who were habitually myopic but rendered near-emmetropic following refractive surgery with those of age-matched controls. Any difference in results between the two cohorts could therefore be confounded by the pre-operative myopia of cases (e.g. myopic eyes may habitually have more HOA’s than age-matched emmetropes and the sensitivity of myopes to blur may be poorer than age-matched emmetropes [26–28]). The second intervention arm addressed this issue by having the pre-operative IQ of subjects act as an internal control for any change in IQ post surgery. A sample size of 28 subjects was recommended in each study arm based on an estimated study power of 80%, a confidence level of 95% and a difference in RMS deviation of HOA’s (HORMS) of 0.35μ between cases and controls. The study protocol adhered to the tenets of declaration of Helsinki. This study was duly approved by the Institutional Review Board of L V Prasad Eye Institute (LVPEI), Hyderabad. All subjects participated in the study after signing a written informed consent form. Pre-operative myopia of cases ranged from -2.0D to -9.0D in both arms of the study. The median (25^th^– 75^th^ IQR) spherical equivalent refraction of the left eye was -6.5D (-3.3D to -7.5D) in the first arm of the study and -5.9D (-3.5D to -7.0D) in the second arm of the study (Sheet 1 in S1 Dataset). All cases underwent wavefront guided LASIK or PRK between August 2012 and June 2014 using the Bausch & Lomb Technolas^®^ 217z Excimer Laser by two surgeons at the Cornea and Refractive Surgery services of LVPEI (one of them is a co-author of this study). For LASIK, the flaps were created on the ZEISS VisuMax^®^ Femtosecond Laser system with a planned flap thickness of 120μ. The ablation zone for both refractive surgery procedures was 6.5mm in all subjects. All cases were aimed to have emmetropic post-operative refraction. There was no planned under-correction of refractive error for any participant in this study. All controls were students or staff of LVPEI and they were all within 0.50D of emmetropia.

HOA’s were measured using the Imagine Eyes^®^ IRX3 wavefront aberrometer after cycloplegia with 1% Cyclopentolate Hydrochloride to ensure stable accommodation and ≥6mm pupil diameter [29]. Technical details and performance evaluation of this aberrometer can be obtained from http://www.imagine-eyes.com/wp-content/uploads/2014/08/M-DCP-001-g-irx3-datasheet.pdf. Three scans were obtained from each subject and they were averaged after scaling each Zernike coefficient to the 6mm pupil diameter using computational techniques described earlier [30]. HORMS was calculated as the square root of sum of squares of 3^rd^ to 8^th^ order Zernike coefficients.

IQ was determined computationally for each subject for 555nm monochromatic light from the individual aberration profiles using standard Fourier optics techniques implemented using custom-written MATLAB^®^ software [21]. IQ was computationally described using the logVSOTF metric, as it has been shown previously to correlate well with high-contrast logMAR acuity for normal and highly aberrated eyes (e.g. Keratoconus) [31–33]. Through-focus analysis was performed using this metric for a range of target vergence –2.5D (hyperopia) to +2.5D (myopia) by systematically changing the defocus term of the Zernike series [Z(2,0)] while leaving the coefficients of all other Zernike terms unchanged. The defocus term was varied in 0.25D steps within ±1D range to ensure fine analysis of IQ change in the two arms of the study and the step size was increased to 0.5D outside of the ±1D range. The computational through-focus curve of each subject was then interpolated in 0.01D steps using a spline interpolation function. The peak IQ of this interpolated function and the dioptric position that corresponded to this peak IQ was then noted. Computational DOF was also determined from this curve as the total range of hyperopic and myopic foci over which the IQ remained above 80% (in log units) of the peak IQ [34]. Other IQ thresholds (e.g. 50% of the peak value [12, 16]) were also attempted on a subset data but the results did not reveal any difference in trends other than an overall expansion (with a more liberal threshold) or contraction (with a more conservative threshold) of the DOF. The final analysis was therefore restricted to the 80% threshold. Similarly, computational analyses were also performed on a subset of data with other IQ metrics (e.g. logVSX or logVSMTF [21]) and the trends were similar to what was obtained with logVSOTF.

Monocular high-contrast (98%) logMAR visual acuity was determined at 3m using COMPlog^®^, a commercial software that randomizes optotype presentation and determines acuity using a staircase thresholding procedure [35]. Briefly, a series of 5 Sloan optotypes were displayed on a LCD screen (1680 x 1050 pixels resolution) and their angular subtense decreased until 3 of 5 optotypes in a given line were incorrectly identified. The procedure automatically terminated when the aforementioned threshold was reached, thereby minimizing any examiner bias in the visual acuity recordings. Distance logMAR acuity was recorded as the total number of optotypes correctly identified at termination, with 0.02 logMAR units allotted per optotype [35]. The acuity measurements were obtained for -2.5D (hyperopia) to +2.5D (myopia) induced refractive errors in 0.25D steps within ±1D range and in 0.5D steps outside of this range to construct the psychophysical through-focus curve for each subject. This curve was then interpolated in 0.01D steps using a spline interpolation function to obtain the peak value of this curve and the best focus where this peak value was achieved. Psychophysical DOF was determined from this curve as the total range of hyperopic and myopic foci over which the logMAR acuity remained above 80% (in log units) of the peak value. All these calculations were identical to what was done for the computational analysis, thereby making the results of the two analyses comparable to each other. All acuity measurements were obtained following cycloplegia and viewing through a 6mm diameter artificial aperture placed in a trial frame at 14mm vertex distance. Decentration of the artificial aperture with respect to the subject’s natural pupil could induce unwanted aberrations and optical distortions during psychophysical testing, especially in eyes undergoing LASER refractive surgery. While it was not possible to achieve perfect alignment between the artificial aperture and natural pupil, alignment was largely ensured through visual inspection of the subject’s eye at random times throughout the experiment and after every break. Subjects were also asked to report if they perceived any misalignment in the aperture position and if they had to adopt any abnormal eye or head position to view the visual targets. Any residual sphero-cylindrical refractive error of the subject (obtained with retinoscopy) was corrected using appropriate trial lenses.

Variations in retinal IQ have been shown to affect spatial resolution with a reduction in the contrast and luminance of target [36]. To determine if the results of our psychophysical experiment may also be dependent on the target contrast used, a first control experiment was performed wherein the main experiment was repeated on a subset of 30 subjects (19 to 26yrs) using low contrast (25%) logMAR acuity targets. All other data collection procedures were identical to the main experiment.

DOF determined using the acuity cut-off in the main experiment represents loss of fine details in the retinal image. While this measure may carry relevance to clinical decision making, it is somewhat removed from the subject’s everyday experience of blur perception [37]. In order to determine the impact of refractive surgery on a more practical and subjective measure of blur “perception”, a second control experiment was performed on 20 cases (22–34yrs) before and after refractive surgery and on 20 emmetropic controls (20–25yrs) where the DOF was measured using three criteria described earlier by Atchison et al: “just noticeable blur” (JNB), “bothersome blur” (BB) and “objectionable blur” (OBB) [37]. Briefly, the subjective DOF was measured using a Badal Optometer set-up with a +5D Badal lens. The distance of the virtual image of 0.3logMAR text was adjusted by moving a -6D auxiliary lens placed between the target and the Badal lens [37]. Movement of the auxiliary lens away from the Badal lens induced hyperopic defocus and measured the negative range of DOF while movement of the auxiliary lens towards the Badal lens induced myopic defocus and measured the positive range of DOF. DOF was quantified as the sum total of the positive and negative range at which the three aforementioned criteria were met [37]. The instruction set for the subjects were identical to that used by Atchison and colleagues. Each measurement was repeated thrice and averaged. All other data collection procedures were identical to the main experiment.

Data was collected from both eyes of all subjects. However, data from only the left eye are reported here. Since the study was not intended to compare the relative performance of LASIK and PRK, data from both procedures were combined and presented together here. Kolmogorov-Smirnov test for normality indicated that other than sphero-cylindrical refractive error and peak logVSOTF, all other data were non-normally distributed. The non-normal distribution of wavefront data is similar to the results of Howland [38] and Salmon and van de Pol [39] but dissimilar from those of Thibos et al [40] who observed most of all individual Zernike modes to follow a normal distribution. Considering the non-normal distribution of our data, all results were described using non-parametric statistics. Mann-Whitney U test was used to test the level significance between two groups (cases vs. controls in first arm and pre-operative vs. post-operative in second arm) in the study. Association between two variables was described using the Spearman Rank order correlation coefficient. Friedman test was performed to compare the three blur criteria based DOF measurements in the second control experiment, followed by the Wilcoxon signed rank test with appropriate Bonferroni correction for pairwise comparison.

## Results

**Fig 1:**
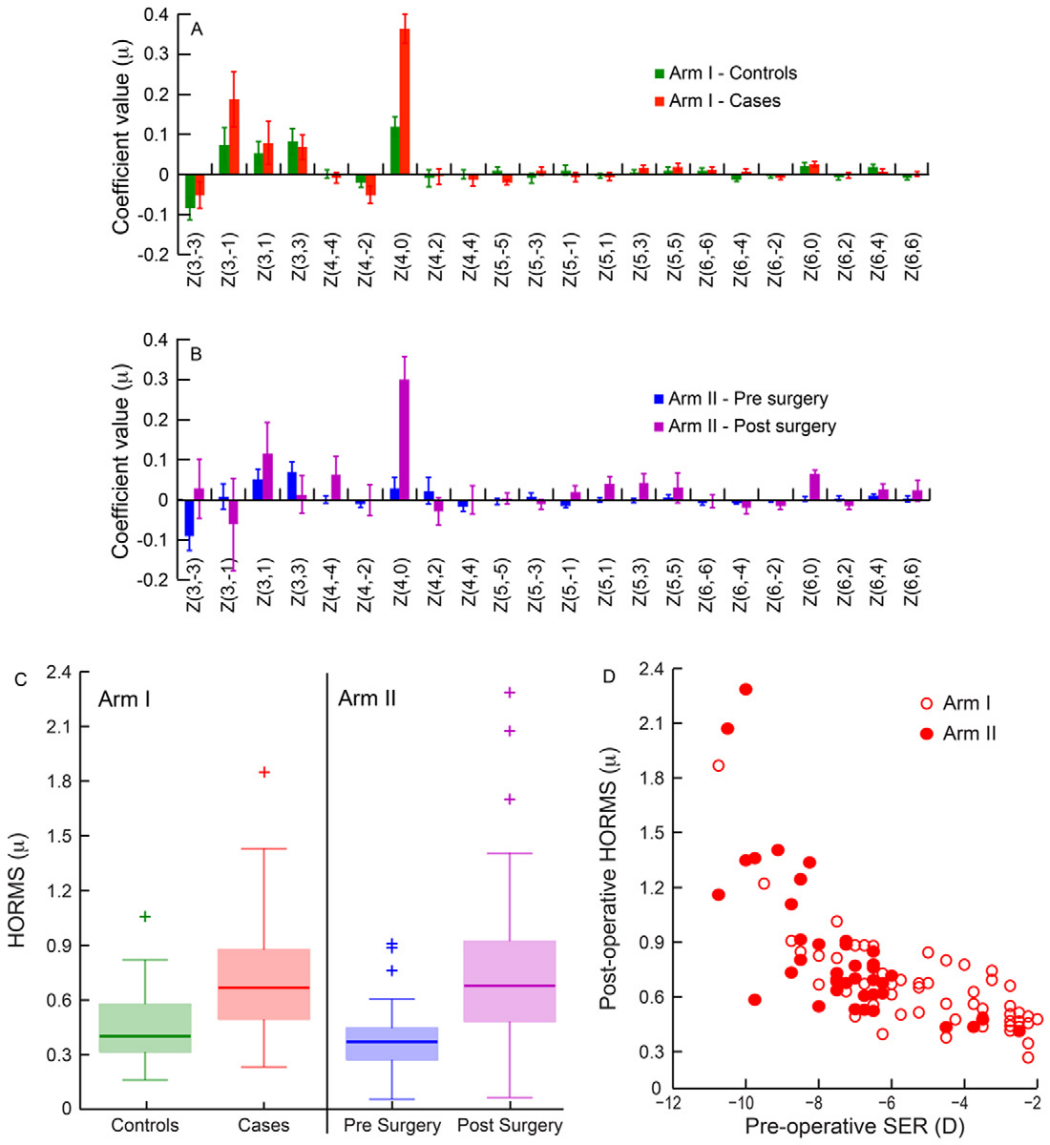
Panels A and B: Histograms showing the pattern of HOA’s observed in the first arm (panel A) and second arm (panel B) of the study. Each bar represents the median (25^th^ and 75^th^ quartiles) coefficients of each order of Zernike polynomial obtained across all subjects in the study. Even though data were obtained up to the 8^th^ order Zernike polynomials, only data up to the 6^th^ order polynomials are shown in this figure. The coefficients of 7^th^ and 8^th^ order polynomials were close to zero in all conditions. Panel C: Box and Whisker plots of the HORMS in the two arms of the study. The solid horizontal line indicates the median HORMS, the bottom and top end of the box indicates the 25th and 75^th^ quartiles, respectively, the error bars indicate the 1^st^ and 99^th^ quartiles, respectively, and the plus symbols show outliers. Panel D: Post-operative HORMS of the subject plotted against their respective pre-operative spherical-equivalent refraction (SER) in the two arms of the study. Negative values along abscissa indicate increasing myopia.

Pre-operative myopia of all cases reduced to within ±0.5D of spherical refractive error and ≤1.0D of astigmatism post-operatively. The subject’s post-operative refractive error correlated only modestly with the magnitude of pre-operative myopia and astigmatism (r = 0.34; p = 0.01 for both). As expected from previous reports [5, 41], of all HOA’s, the coefficients of horizontal coma [Z(3,1)], vertical coma [Z(3,-3)] and primary spherical aberration [Z(4,0)] were higher in cases than in age-matched controls in the first arm of the study (Fig 1A and Sheet 2 in S1 Dataset) and it increased following refractive surgery in the second arm of the study (Fig 1B and Sheet 3 in S1 Dataset). The median [25^th^ to 75^th^ inter-quartile range (IQR)] HORMS of cases [0.65μ (0.59 to 0.88μ)] was statistically significantly larger than those of controls [0.35μ (0.30 to 0.48μ)] in the first arm of the study (z = 3.7; p<0.002) (Fig 1C and Sheet 4 in S1 Dataset). The post-operative HORMS of subjects who underwent refractive surgery [0.64μ (0.51 to 0.89μ)] were statistically significantly larger than their pre-operative values [0.37μ (0.27 to 0.43μ)] in the second arm of the study (z = 5.39; p<0.001) (Fig 1C and Sheet 4 in S1 Dataset). Even while the median HORMS of those who underwent refractive surgery were similar in the two arms of the study, there was larger intersubject variability in HORMS in the second arm compared to the first arm, as evidenced by the larger interquartile range and number of outliers in the two groups (Fig 1C). There was a statistically significant positive correlation between the pre-operative spherical equivalent of refraction and the post-operative HORMS across both arms of the study (r = 0.68; p<0.0001), indicating that subjects with greater magnitude of per-operative myopia experienced higher HOA’s post-operatively (Fig 1D). Pre-operative HORMS of all subjects were very similar [Median (25^th^ to 75^th^ IQR): 0.37μ (0.26 to 0.42μ)] and poorly correlated with the post-operative HORMS (r = 0.2; p = 0.19), suggesting that the surgical procedure was largely responsible for the increased HOA’s experienced post-operatively.

**Fig 2:**
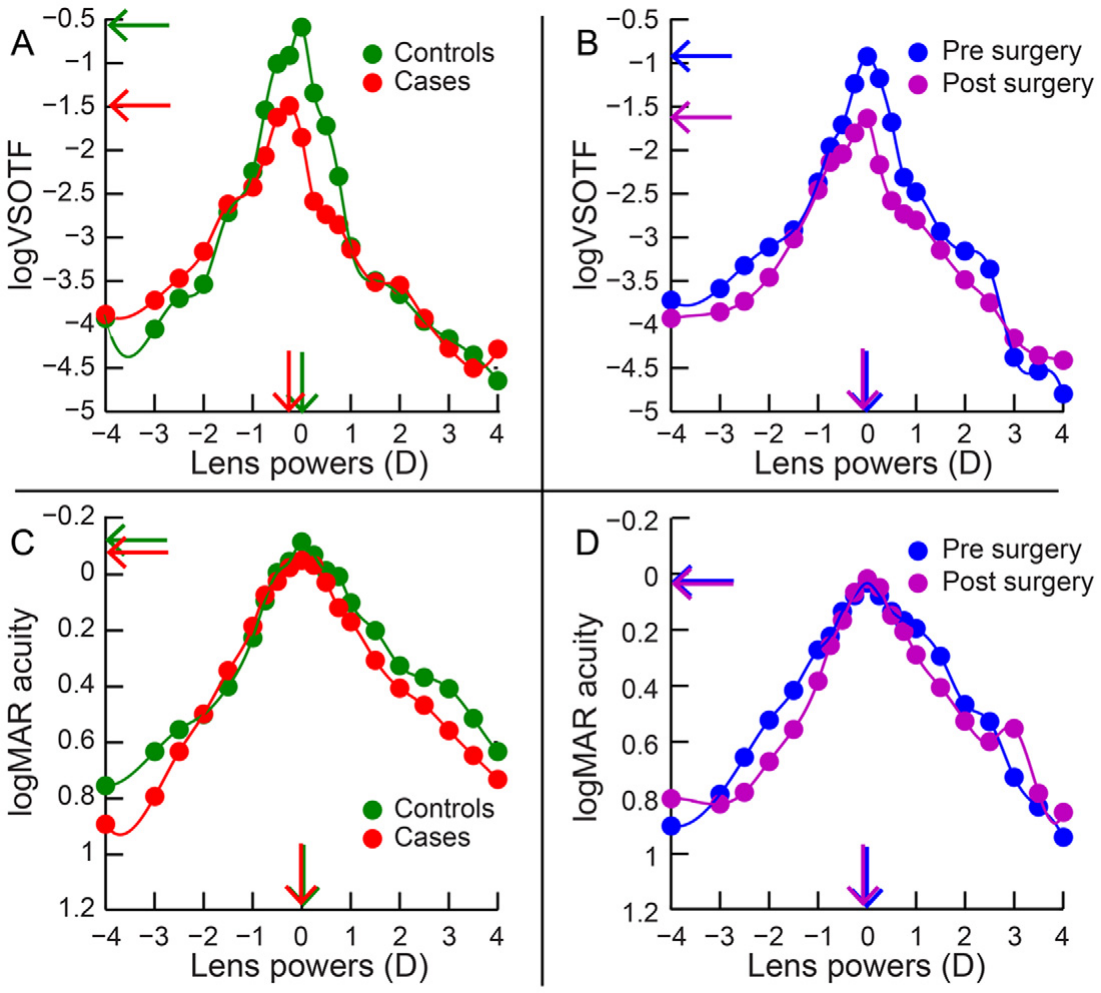
Average computational (panels A and B) and psychophysical (panels C and D) through-focus curves of all subjects obtained by plotting logVSOTF or logMAR acuity for each induced myopic and hyperopic lens power The solid circles indicate individual data points while the curve indicate the spline fit to the data. Panels A and C show through-focus curves for the first arm of the study while panels B and D show through-focus curves for the second arm of the study. Horizontal and vertical arrows in each panel indicate peak IQ and best focus location, respectively.

**Fig 3:**
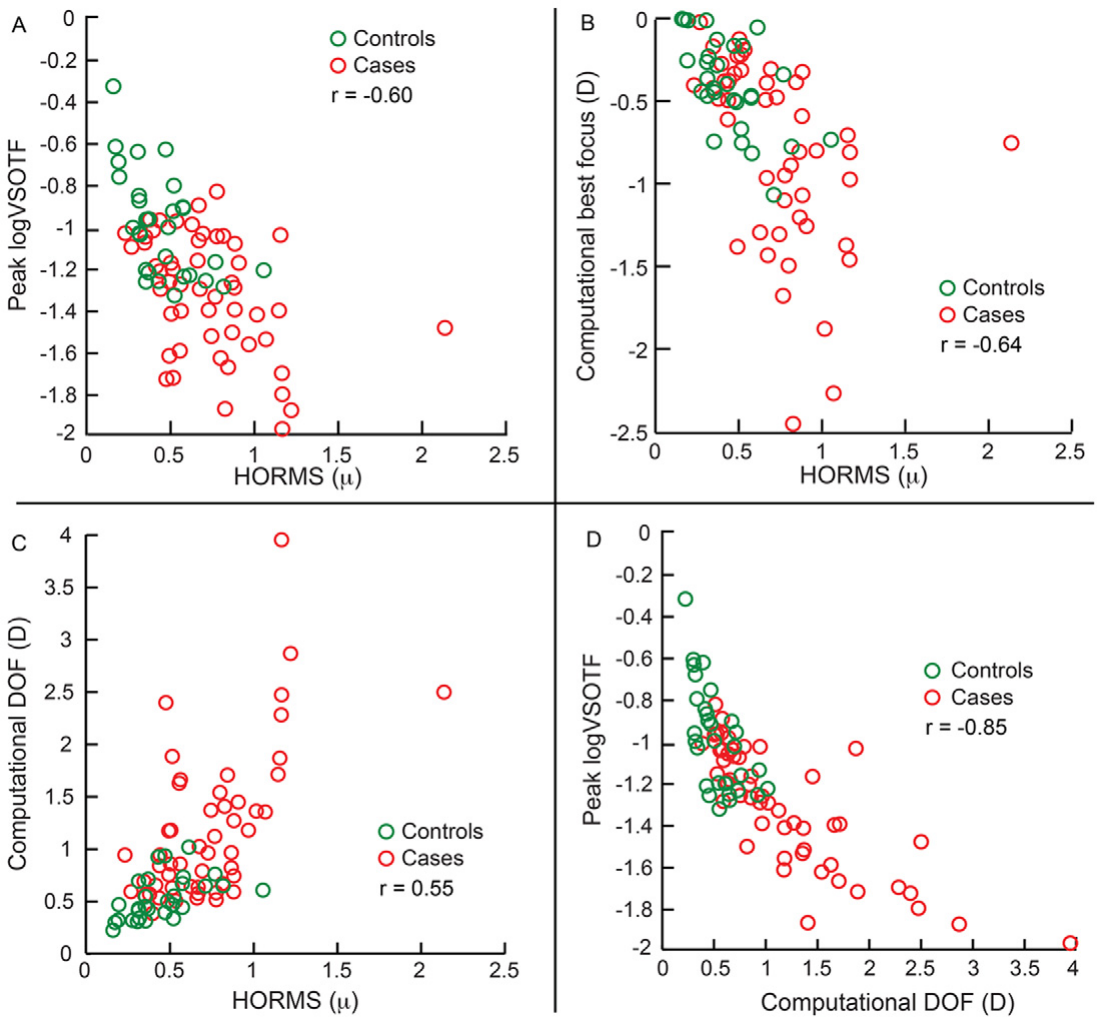
Relation between peak logVSOTF, computational best focus, computational DOF and HORMS obtained from the first arm of the study. Panels A to C show data of peak logVSOTF, computational best focus and the computational DOF of both controls and cases plotted against their HORMS values, respectively. Panel D shows data of peak logVSOTF values plotted against the respective computational DOF.

**Fig 4:**
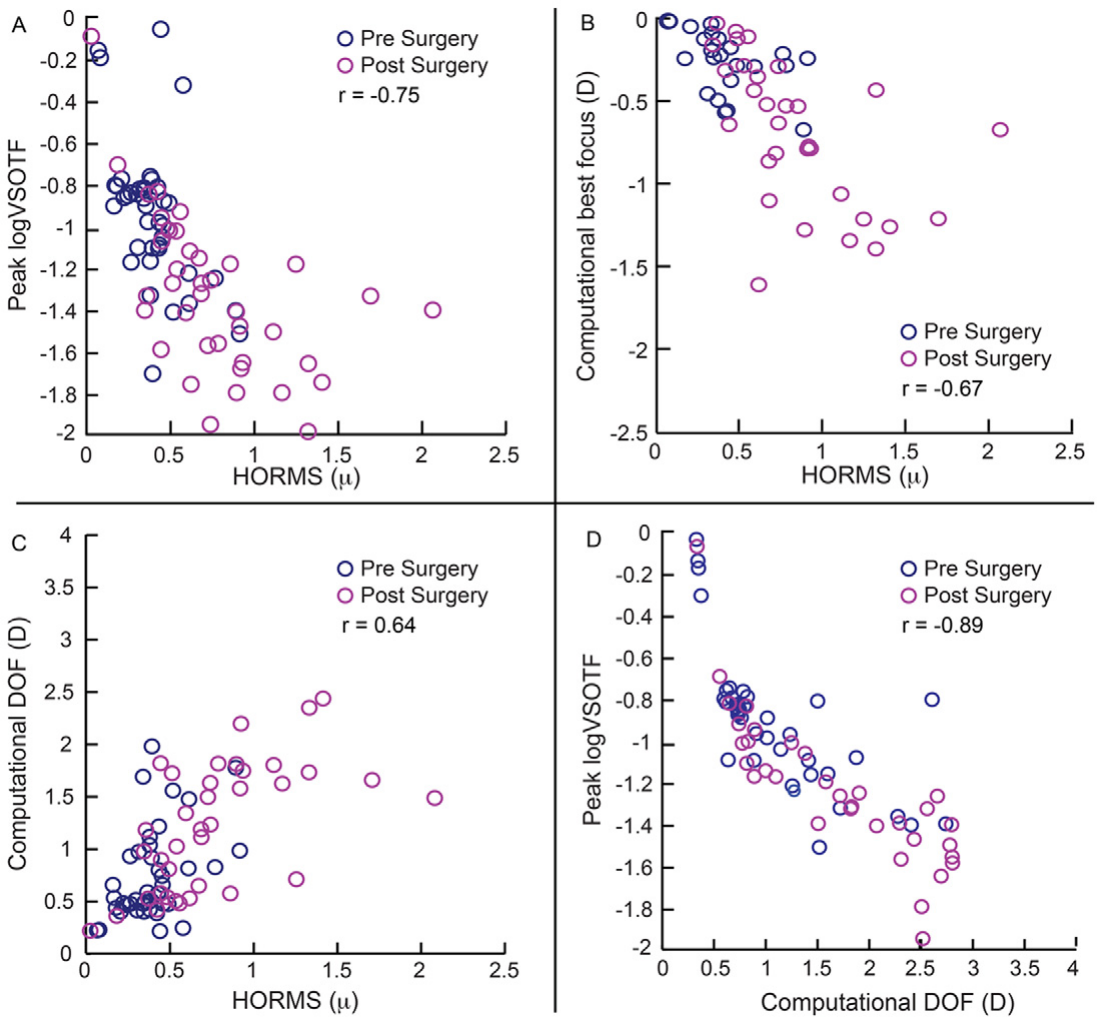
Relation between peak logVSOTF, computational best focus, computational DOF and HORMS obtained from the second arm of study. All other details are similar to Fig 3.

Fig 2 shows the average computational (panels A and B) and psychophysical (panels C and D) through-focus curves of all subjects obtained from the first and second arms of the study (see Sheets 5, 6, 9 and 10 in S1 Dataset for defocus curves of individual participants). In the first arm, the peak logVSOTF was more negative in cases [Median (25^th^ to 75^th^ IQR): -1.39 (-1.66 to -1.16)] than in controls [-1.02 (-1.26 to -0.79)] (z = -4.7; p<0.001), indicating a loss of peak IQ following refractive surgery (Fig 3A and Sheet 7 in S1 Dataset). The magnitude of loss in peak logVSOTF increased with the magnitude of HORMS across all cases and controls (r = -0.6; p<0.001) (Fig 3A, Table 1). The computational best focus was shifted more myopic in cases [−0.48D (−1.01 to −0.31D)] than in controls [-0.38D (-0.50 to -0.04D)] (z = -2.5; p<0.01), with this shift being significantly negatively correlated with the magnitude of HORMS (r = -0.64; p<0.001) (Fig 3B, Table 1 and Sheet 7 in S1 Dataset). The computational best focus was also modestly but statistically significantly correlated with the magnitude of primary spherical aberration [Z(4,0)] across cases and controls in the first arm of the study (r = -0.52; p<0.001) (data not shown). The computational DOF [1.18D (0.75 to 1.70D)] of cases was statistically significantly larger than those of controls [0.63D (0.44 to 0.93D)] (z-4.8; p<0.001), with these values being positively correlated with the magnitude of HORMS (r = 0.55; p = <0.001) (Fig 3C, Table 1 and Sheet 7 in S1 Dataset). As expected, the peak logVSOTF and the computational DOF were also significantly negatively correlated with each other (r = -0.85; p<0.001) (Fig 3D, Table 1). For cases, the post-operative peak logVSOTF was significantly negatively correlated with the magnitude of pre-operative myopia (r = -0.55; p<0.001) while the computational DOF was significantly positively correlated with the magnitude of pre-operative myopia (r = 0.6; p<0.001). The results of the second arm were basically very similar to the first arm (Fig 4, Tables 1 and 2 and Sheet 8 in S1 Dataset). Unlike the first arm, the magnitude of primary spherical aberration before and after surgery or the change in primary spherical aberration with surgery was not significantly correlated with computational best focus (r≤0.2; p≥0.2; data not shown) even though HORMS was significantly correlated with computational best focus (Fig 4B; Table 1).

**Table 1:** Results of the computational analyses performed in the first and second arms of the study. Spearman’s rank correlation coefficient and the corresponding p-value obtained between the outcome variables used in this study.

	r-value	p-value
**Case-Control arm of the study**		
Peak logVSOTF Vs. HORMS	-0.6	<0.001
Computational best focus Vs. HORMS	-0.64	<0.001
Computational DOF Vs. HORMS	0.55	<0.001
Peak logVSOTF Vs. Computational DOF	-0.85	<0.001
**Intervention arm of the study**		
Peak logVSOTF Vs. HORMS	-0.75	<0.001
Computational best focus Vs. HORMS	-0.67	<0.001
Computational DOF Vs. HORMS	0.64	<0.001
Peak logVSOTF Vs. Computational DOF	-0.89	<0.001

**Table 2:** Results of the psychophysical analyses performed in the first and second arms of this study. All details are same as Table 1.

	r-value	p-value
**Case-Control arm of the study**		
Peak logMAR acuity Vs. HORMS	0.22	0.01
Psychophysical best focus Vs. HORMS	0.02	0.84
Psychophysical DOF Vs. HORMS	-0.06	0.55
Peak logMAR acuity Vs. Psychophysical DOF	-0.27	0.01
**Intervention arm of the study**		
Peak logMAR acuity Vs. HORMS	-0.07	0.31
Psychophysical best focus Vs. HORMS	-0.04	0.8
Psychophysical DOF Vs. HORMS	0.16	0.16
Peak logMAR acuity Vs. Psychophysical DOF	-0.10	0.53

**Fig 5:**
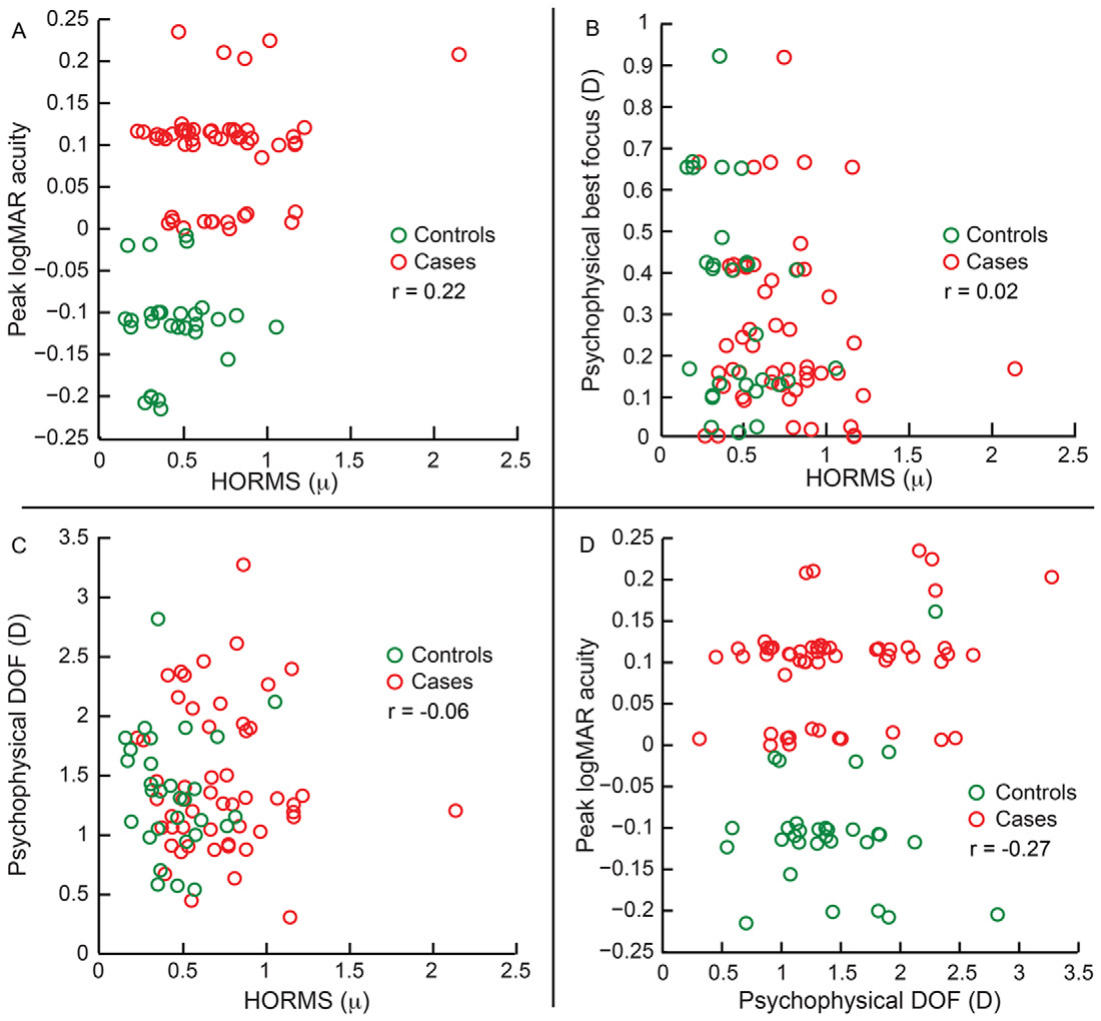
Relation between peak high contrast logMAR acuity, psychophysical best focus, psychophysical high contrast DOF and HORMS obtained from the first arm of the study. All other details are similar to Fig 3.

**Fig 6:**
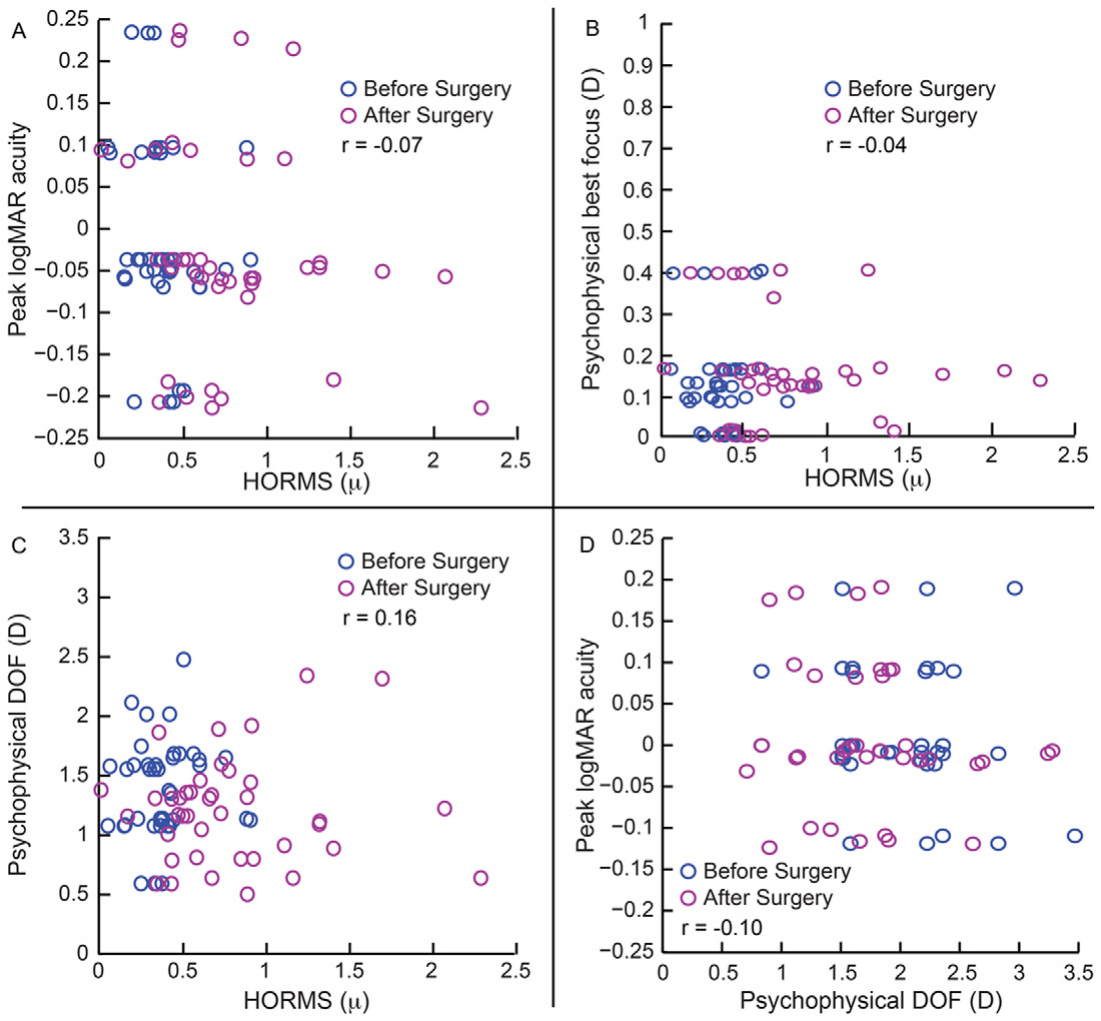
Relation between peak high contrast logMAR acuity, psychophysical best focus, psychophysical high contrast DOF and HORMS obtained from the second arm of the study. All other details are similar to Fig 5.

The median high-contrast logMAR acuity [0.1 logMAR (0.06 to 0.11 logMAR)] of cases was statistically significantly poorer than those of controls [-0.10 logMAR (-0.13 to -0.10 logMAR)] (z>7.2; p<0.001) and these were poorly correlated with the subject’s HORMS values (r = 0.22; p = 0.01) (Fig 5A, Table 2 and Sheet 11 in S1 Dataset). The psychophysical best focus [0.10D (-0.15 to 0.16D)] and the psychophysical DOF [1.32D (1.22 to 1.79D)] of cases were not statistically significantly different from those of controls [psychophysical best focus: -0.14D (-0.4 to 0.15D); psychophysical DOF: 1.23D (1.12 to 1.63D)] (z<-0.06; p>0.59) and were also poorly correlated with the eye’s HORMS (r≤|0.06|; p>0.54) (Fig 5B & 5C, Table 2 and Sheet 11 in S1 Dataset). The psychophysical best focus was also poorly correlated with the magnitude of primary spherical aberration (r = 0.12; p = 0.42) (data not shown). The peak logMAR acuity was also not correlated with the psychophysical DOF (r = -0.27; p = 0.01) (Fig 5D, Table 2). Like the computational data, the psychophysical results obtained in the second arm of the study were also quite similar to those obtained from the first arm (Fig 6, Table 2 and Sheet 12 in S1 Dataset for details). Overall, the psychophysical and computational data obtained in here differed in that none of the acuity-related outcome variables were correlated with the magnitude of HOA’s experienced by the subject. There was also poor correlation between the peak high-contrast logMAR acuity and peak logVSOTF values obtained in both arms of the study (|r|≤0.3; p>0.1 for both). This result is in line with the results of Applegate et al who observed poor correlation between high contrast logMAR acuity and IQ metrics for those with really good logMAR acuities (20/17 or better) [36].

**Fig 7:**
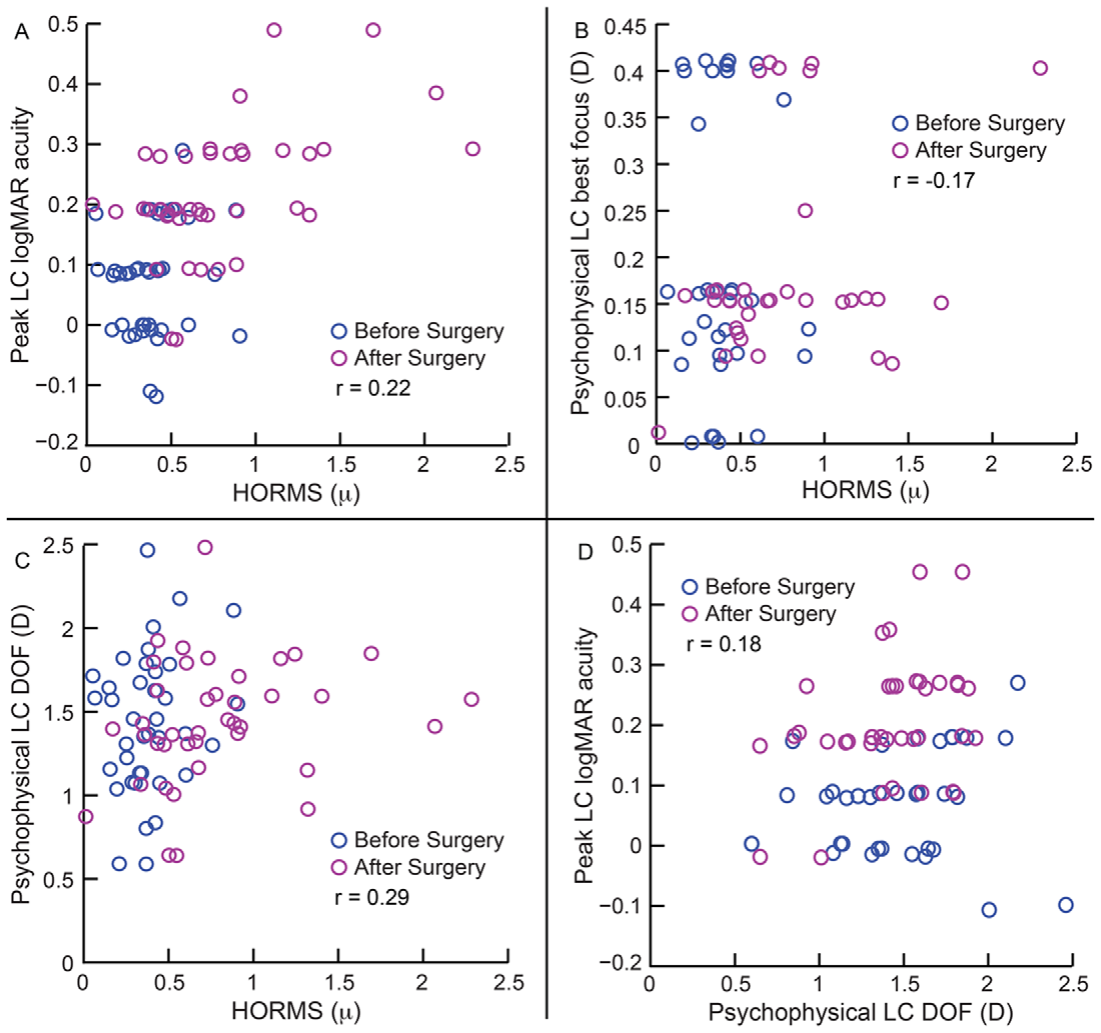
Relation between logMAR acuity, psychophysical best focus, psychophysical DOF and HORMS obtained from first control experiment. All other details are similar to Fig 5.

The median post-operative low contrast logMAR acuity in the first control experiment [0.20logMAR (0.20 to 0.30logMAR)] was statistically significantly poorer than their pre-operative values [0.10logMAR (0.10 to 0.13logMAR)] (z = -6.2,p<0.001) (Fig 7 and Sheet 13 in S1 Dataset). The remainder of results were however very similar to the main experiment in that the median post-operative psychophysical low contrast best focus [-0.09D (-0.16 to 0.15D)] and the psychophysical low contrast DOF [1.56D (1.34 to 1.62D)] were not statistically significantly different from the pre-operative values [psychophysical low contrast best focus: -0.09D (-0.16 to 0.41D); psychophysical low contrast DOF: 1.56D (1.23–1.75D)] (z<-0.65; p>0.27) (Fig 7 and Sheet 13 in S1 Dataset). The correlation coefficients between the low contrast acuity parameters and HORMS were also not statistically significant (r≤0.29; p≥0.17 for all) (Fig 7, Table 3).

**Table 3:** Results of the analyses performed in first control experiment of this study. All details are same as Table 1.

	r-value	p-value
Peak low contrast logMAR acuity Vs. HORMS	0.22	0.17
Psychophysical best focus for low contrast logMAR acuity Vs. HORMS	-0.17	0.27
Psychophysical low contrast DOF Vs. HORMS	0.29	0.07
Peak low contrast logMAR acuity Vs. Psychophysical DOF	0.18	0.27

**Fig 8:**
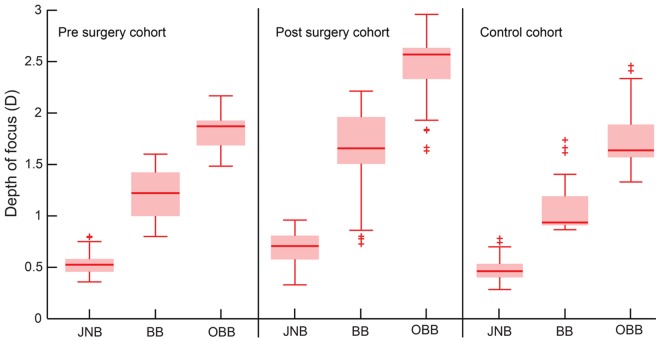
Box and whisker plots of the DOF obtained from the second control experiment. Data obtained from the three different DOF criteria are shown in each panel. Details of the box and whisker plot are similar to Fig 1C.

In the second control experiment, the median DOF after refractive surgery was larger than those before surgery and in age-matched controls, with the difference being largest for the OBB criterion followed by the BB and the JNB criteria [Χ^2^(2) = 217.9; p<0.001] (Fig 8 and Sheet 14 in S1 Dataset). There was a statistically significant difference between all three DOF criteria before and after surgery (z = -5.5; p<0.001 for all) and also between age-matched controls and the post-operative cohort (z = -4.7; p<0.001 for all). There was however no statistically significant correlation between the HORMS and the three DOF criteria in the pre-operative cohort (r≤-0.03; p = 0.87 for all), post-operative cohort (r≤-0.33; p = 0.35 for all) and for age matched controls (r≤-0.22; p = 0.28 for all).

## Discussion

This study prospectively evaluated the impact of increased HOA’s on the monocular IQ of subjects undergoing wavefront optimized LASIK and PRK procedures for the treatment of myopia. The key results of the study are as follows:

1. As previously reported [2–5], the HORMS for 6mm pupil diameter was higher in cases than in age-matched controls and higher after refractive surgery than before surgery (Fig 1A–1C). While the pre-operative HORMS was similar across all subjects, the post-operative HORMS increased with the magnitude of pre-operative myopia (Fig 1D).
2. Increase in HORMS was associated with a reduction in peak logVSOTF, a myopic shift in computational best focus and a widening of the computationally derived DOF in both arms of the study (Figs 3A–3C and 4A–4C). The widening of computational DOF was also associated with a drop in peak logVSOTF (Figs 3D and 4D).
3. None of the IQ parameters derived psychophysically from high- and low-contrast logMAR acuity correlated well with the magnitude of HORMS in subjects who participated in this study (Figs 5–7).
4. Median DOF obtained using the dioptric point of first blur perception was larger after refractive surgery than before surgery and in age-matched controls (Fig 8). The DOF was however poorly correlated with the HORMS even in this experiment.

The results of this study have important implications for the clinical management of patients following refractive surgery. First, the similarity between the case-control and intervention arms of this study indicated that both study designs are equally effective at investigating the impact of eye’s optics on visual performance following LASER refractive surgery. These results are expected because of the similarity in subject profiles (e.g. age, magnitude of myopia), surgical procedure and experimental protocols used for outcomes evaluation. The good correlation between post-operative HORMS and pre-operative myopia indicates that eyes with greater degrees of myopia are likely to experience more degradation in their optics following refractive surgery than those with smaller degrees of myopia (Fig 1D). These results are expected because the correction of higher myopia requires greater flattening of the central cornea, leading to increased values of HOA’s (especially, spherical aberrations) in these eyes [42, 43].

The small but significant myopic shift in computational best focus indicates that post-operative best IQ may not be achieved at emmetropic refractions but at focal lengths that are closer than optical infinity (Figs 3B & 4B). These results are largely expected from the increase in the magnitude of positive spherical aberration [Z(4,0)] following refractive surgery in both arms of the study (Fig 1A & 1B). An increase in positive spherical aberration implies that the marginal light rays entering the pupil tend to focus more myopically than paraxial light rays entering through the center of the pupil. The “circle of least confusion” for positive spherical aberration or the spherical power that optimizes image quality in the VSOTF metric therefore tends to shift myopically with an increase in the magnitude of positive spherical aberration following LASER refractive surgery [13, 44, 45]. This effect is supported by the significant correlation between the magnitude of spherical aberration and the computational best focus in the first arm of the study (r = -0.52). This effect is also expected to be greater in patients with larger magnitudes of post-operative HOA’s, when the pupil diameters are large (e.g. mesopic or scotopic light levels relative to photopic light levels) and it may also account for some of the discrepancy between objective and subjective refractions observed after refractive surgery [46, 47]. The role of spherical aberrations have also been implicated in observed discrepancy between objective and subjective methods of recording the eye’s accommodative state [25]. Such artifactual difference may be exaggerated in eyes undergoing refractive surgery because of the increased magnitude of positive spherical aberration in these eyes.

The widening of post-operative computational DOF and the commensurate loss in peak IQ indicate that the relatively sub-standard IQ now exists over a larger dioptric range in patients after refractive surgery (Figs 3D & 4D). Manipulating HOA’s to expand the DOF is the central strategy of many multifocal contact lenses and intraocular lenses to improve the range of useful intermediate and near vision of presbyopes and pseudophakes, albeit with a small loss to distance acuity [48–51]. In this context, patients who have undergone LASER refractive surgery achieve the same optical effect of a multifocal lens in that their expanded DOF might also support useful intermediate and near vision without exerting much accommodative effort—a scenario that is useful with the onset of presbyopia. However, this might pose a challenge to the binocular near vision in pre-presbyopic ages as the demands on accommodation and its coupled vergence response may be altered due to modification in the eye’s DOF [22–25]. This issue needs further exploration.

DOF obtained using the “blur perception” criteria did show an increase after refractive surgery, although there was still no correlation with the magnitude of HOA’s (Fig 8). These criteria represent a more practical scenario of how blurred vision may be “perceived” in everyday situations as opposed to the acuity cut-off criterion that specifically represents a loss of fine details in the retinal image [37]. These results indicate that changes in DOF following refractive surgery may depend on how DOF is measured—a technique relying on subjective impression of blur may be more sensitive to changes in IQ after refractive surgery than those tracking changes in logMAR acuity to measure DOF. These results further imply that subject’s conscious awareness or perception of blur may be diminished after LASER refractive surgery (hence translating into larger subjective DOF), even while their ability to resolve fine details may remain largely unaltered.

The psychophysical data obtained in this study were different from the computational data obtained on the same subject and also different from previous reports on changes in high-contrast logMAR acuity and DOF with induced HOA’s [7, 12, 14, 15]. A combination of six different factors may account for this difference. First, the lack of correlation between psychophysical IQ and HOA’s in the present study could be because the magnitude of aberrations induced after LASER refractive surgery were smaller than what would cause a significant deterioration in high contrast logMAR acuity [6, 52]. This explanation is unlikely for the HOA’s induced in these previous studies were lesser than or similar in magnitude of HOA’s experienced here [7, 12, 14, 15]. The visual system, in general, must therefore be sensitive to the magnitude of alteration in optics experienced by our subjects in this study.

Second, the lack of correlation may be because of the sensitivity of experimental measure used in this study (i.e. high contrast logMAR acuity). This possibility follows previous observations of a minimal change in high-contrast logMAR acuity and a small but significant loss of low contrast (10% to 18%) acuity following LASER refractive surgery [53, 54]. The correlation between IQ metrics and logMAR acuity also drop significantly for logMAR acuity measured at high contrast and photopic light levels, vis-à-vis, low contrast and mesopic or scotopic light levels [36]. In fact, target contrast had to reduce to about 5% before perceptible changes in logMAR acuity could be seen in otherwise normal individuals [55]. Perhaps, the psychophysical measures of visual performance would have better correlated with the magnitude of optical degradation for contrast levels lower than what was tested here (~100% and 25% for high and low contrast logMAR acuity, respectively). As a related issue, logMAR acuity was obtained in this study using a psychophysical staircase paradigm with discrete steps in the logMAR acuity scale [35]. This was different from some previous studies wherein logMAR acuity was calculated using psychometric functions of percent current response versus letter size that contained much finer steps of logMAR scale [7, 44]. The coarse quantization of logMAR acuity in the current study may have resulted in poor correlation of the psychophysical data with the magnitude of optical degradation. Obtaining logMAR acuity using a psychometric curve, on the other hand, is time intensive and was impractical considering the protocol of the current study.

Third, the difference in psychophysical and computational results may be inherent to the way IQ is computed using the VSOTF metric [21]. VSOTF describes IQ by weighting the optical transfer function (OTF) of a given eye by the neural contrast sensitivity function (CSF) [21]. While the OTF component of the VSOTF metric represents the loss of image fidelity experienced uniquely by each eye due to its HOA’s, the weighted neural CSF is constant across all subjects and it does not capture the uniqueness of the subject’s neural visual system [21]. This may specifically be the case in subjects undergoing refractive surgery wherein the CSF may have been altered following prolonged exposure to the novel pattern of HOA’s (see also point six below) [56, 57]. Such alternations in CSF remain unaccounted for in the VSOTF calculations. Psychophysical measures of IQ obtained in this study, however, reflect changes in both the eye’s optics and the neural CSF and may therefore not match the corresponding computational results. An ideal comparison to the psychophysical data would be a computational metric that uniquely represents both the optics and the neural transfer function of the individual being evaluated. Such a metric, however, still does not exist to the best of our knowledge. On a related note, even while the VSOTF metric is well correlated with logMAR acuity in highly aberrated eyes (e.g. Keratoconus [32, 33]), a systematic evaluation of the accuracy and precision of IQ parameters obtained using this metric has not been performed thus far for these eyes. It is therefore possible that the VSOTF performed sub-optimally in some subjects leading to significant intersubject variability in the results, adding to the discrepancy between the computational and psychophysical results (Figs 3–7).

Fourth, all computational measures of IQ obtained in this study were based on monochromatic light of 555nm while all psychophysical measurements were obtained for polychromatic light. The impact of the eye’s chromatic aberrations on visual performance was therefore not captured veridically in the computational measurements and this could partly explain the difference in results obtained in the computational and psychophysical arms of this study. A future study could assess the similar in polychromatic IQ to measures of psychophysical visual performance [44, 58].

Fifth, the observed difference between our psychophysical results and those of previous studies may be related to the way in which HOA’s were induced in previous studies [7, 12, 15]. Most previous studies that have investigated the impact of HOA’s on logMAR acuity have done so by inducing only one or a combination of two Zernike modes of HOA’s at any given point of time [7, 12, 15]. This is different from subjects who underwent LASER refractive surgery wherein there is an increase in several Zernike modes of HOA’s at the same time, which could all interact with each other in complex ways to improve or deteriorate visual acuity [6, 59]. The results of previous literature inducing a limited number of Zernike modes of HOA’s to study visual performance may therefore not be directly related to the present results seen following refractive surgery.

The sixth explanation is related to the duration of exposure to HOA’s in the previous studies, vis-à-vis, those who undergo LASER refractive surgery in the current study. HOA’s were purposefully induced for short periods of time (typically, few minutes) in the previous studies and they are all likely to return to their “baseline” optical state upon completion of the experiment [7, 12, 15]. Subjects undergoing LASER refractive surgery however experience the impact of increased HOA’s on a more permanent basis and may never return to their pre-operative state [2, 3]. An exposure to increased HOA’s for extended durations may trigger a neural adaptive response that optimizes the visual experience of the subject to the novel pattern of optical degradation. Such adaptive responses are routinely noticed in patients who change their sphero-cylindrical spectacles and adaptation has also been demonstrated for novel patterns of induced lower- and higher-order wavefront aberrations [56, 57]. In the present study, IQ was assessed 1-month after surgery and the data obtained may therefore represent the “adapted” state of the visual system that has already optimized its spatial visual performance for the novel pattern of aberrations. This is unlikely to be the case with transiently induced HOA’s and hence their impact on spatial vision could have been obvious in previous studies. Although practically challenging, an assessment of IQ and visual performance immediately after refractive surgery might yield results similar to those obtained with induced HOA’s.

In conclusion, the increase in HOA’s experienced after refractive surgery results in a degradation of peak IQ and a persistence of this sub-standard IQ over a larger dioptric range when compared to age-matched control eyes or when compared to the same eyes before surgery. Such an increase in optical degradation however appears to have only a minimal impact on psychophysical estimates of spatial visual performance (high- and low-contrast logMAR acuity and depth-of-focus). The apparent discrepancy between computational and psychophysical results may arise from a combination of several factors that range from limitations in the computational IQ metrics to the sensitivity of psychophysical measures used here to neural recalibration for optimizing spatial visual performance.

## Supporting Information

S1 Dataset Microsoft Excel^®^ file containing raw data of the different analysis performed in this study. This file contains 14 different sheets each containing data from the main computational and psychophysical experiments and the two control experiments performed in this study.
(XLSX)
